# Supplementary material for: Mild replication stress causes chromosome mis-segregation via premature centriole disengagement
Source: Nat Commun. 2019 Aug 8;10:3585. doi: 10.1038/s41467-019-11584-0 (PMC6687892; doi:10.1038/s41467-019-11584-0)
Supplement: Supplementary file 1 — Supplementary Data [file 41467_2019_11584_MOESM1_ESM.pdf]

Supplementary Information

Mild Replication Stress causes Chromosome Mis-segregation via Premature Centriole Dis-engagement

Wilhelm *et al.*

Supplementary Figure 1; Wilhelm et al.

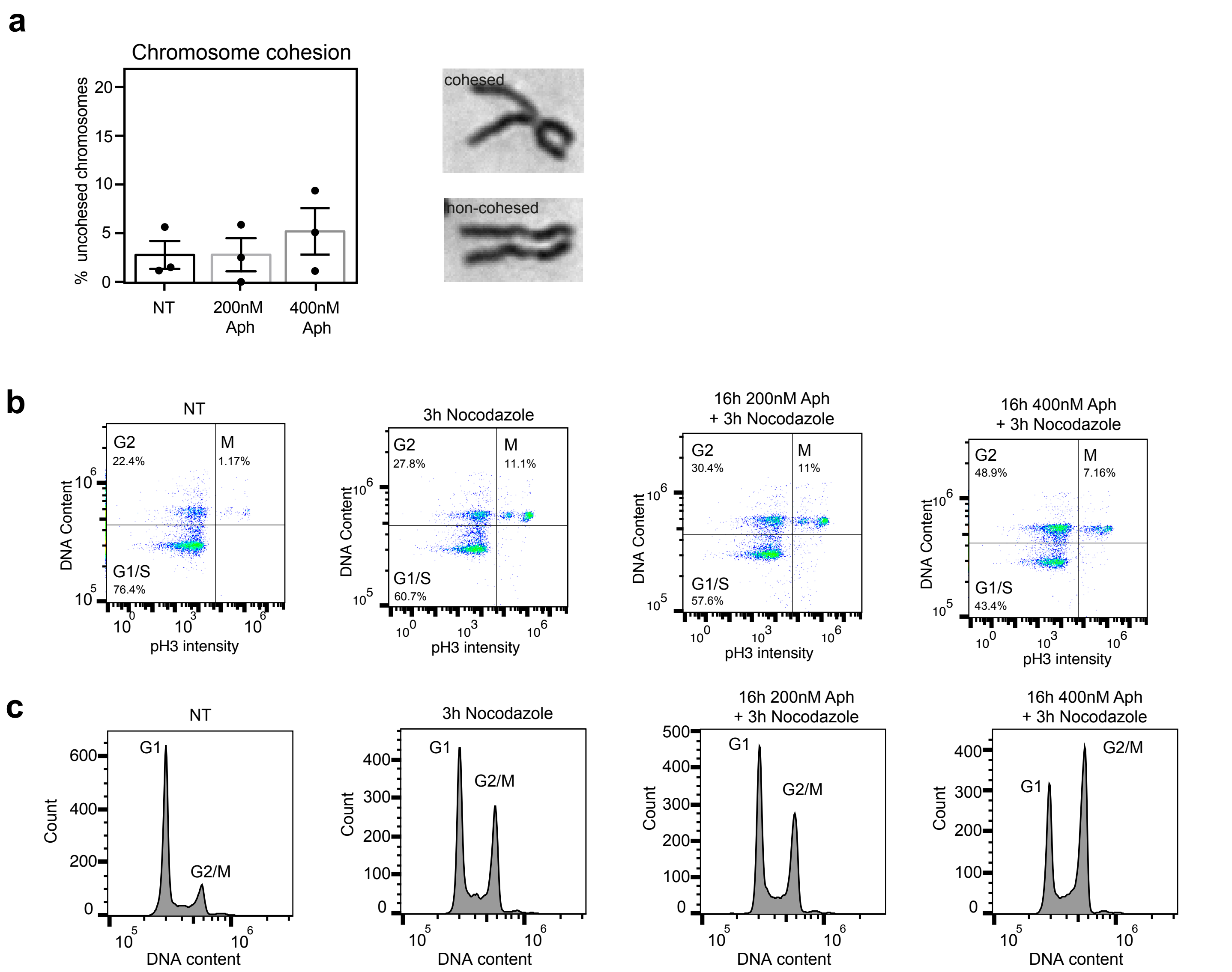

Supplementary Figure 1. Mild replication stress leads to a G2 delay but does not affect chromosome cohesion (**a**) Quantification of non-cohesed chromosomes in RPE1 after no treatment, 200 or 400 nM Aphidicolin. N=3 examining NT=222 metaphase spreads, 200nM Aph=212 and 400nM Aph=212; error bars indicate sem; p = 0,6 in one-way Anova test (NT vs 200 nM Aph p = 0,9999; NT vs 400 nM Aph p = 0,5023). Chromosomes were visualized by metaphase spreads as shown in Figure 1D. Top panel shows an example of a cohesed chromosome, bottom panel of a non-cohesed chromosome.(**b**) Cell cycle profile of RPE1 cells stained with Propidium Iodide (DNA content marker) and phosho-H3 antibody (mitosis marker). Cells were either not treated (NT), treated for 3 hours with 1μM nocodazole, treated for 16 hours with 200 nM Aphidicolin + 1μM nocodazole during the last 3hours of Aphidicolin treatment, or treated for 16 hours with 400 nM Aphidicolin + 1μM nocodazole during the last 3hours of Aphidicolin treatment. The 3 hour nocodazole treatment served as a mitotic trap to reveal the number of cells entering in mitosis. At the end of the 16-hour slot cells were collected, fixed and stained. The cell cycle phase of each population is marked on each panel. (**c**) Histograms of cell cycle profile analysis by FACS of cells shown in panel A. Each peak represents a cell-cycle phase marked on each panel.

Supplementary Figure 2; Wilhelm et al.

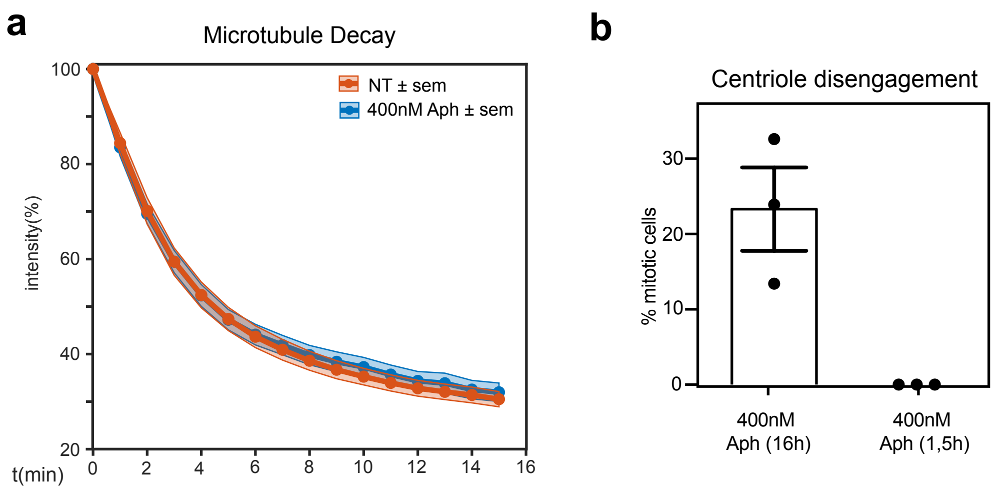

Supplementary Figure 2. Short treatment with Aphidicolin does not induce centriole disengagement and does not increase microtubule stability: (**a**) Quantification of the spindle intensity over time after a nocodazole pulse, as shown in Figure 2, in metaphase RPE1 cells either non-treated or treated with 400nM Aph for 1.5h; N=34 cells/condition. (**b**) Mean percentage of RPE1 cells with multipolar spindles either with 400nM Aph for 16 or 1.5h; note that we did not see a single cell with dis-engaged centrioles after 1.5hrs Aphidicolin; N=3 examining NT=158 mitoses and 400nM Aph(1.5h)=150; error bars represent sem.

Supplementary Figure 3; Wilhelm et al.

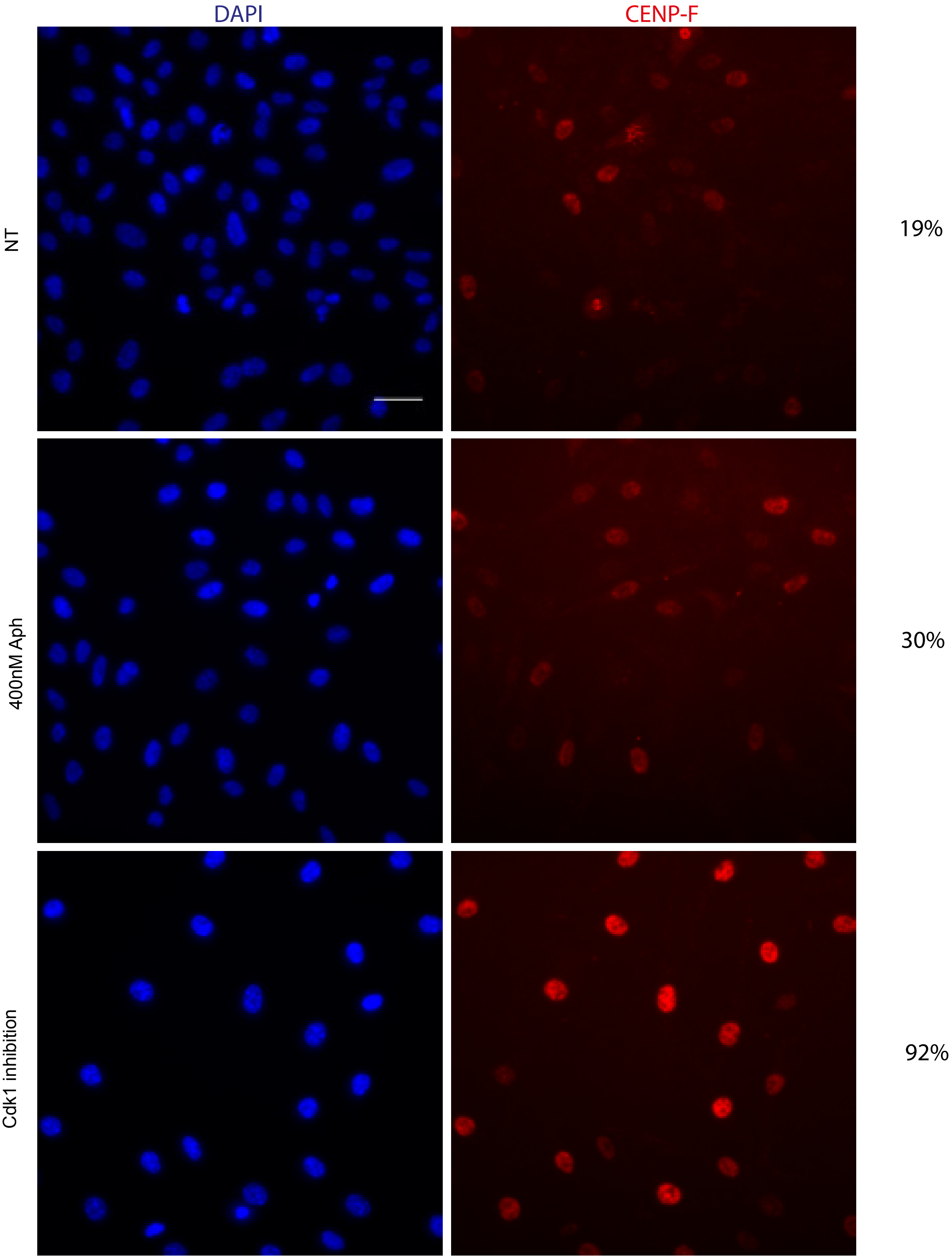

Supplementary Figure 3. Mild replication stress leads to a G2 delay: Representative images of hTert-RPE1 cells after no treatment (NT; top), 400 nM Aphidicolin (middle) or Cdk1 inhbition (bottom) stained with CENP-F antibodies (G2 marker, red) and DAPI (blue). Numbers indicate the percentage of CENP-F positive cells in the corresponding image. Scale bar = 20 μm.

Supplementary Figure 4; Wilhelm et al.

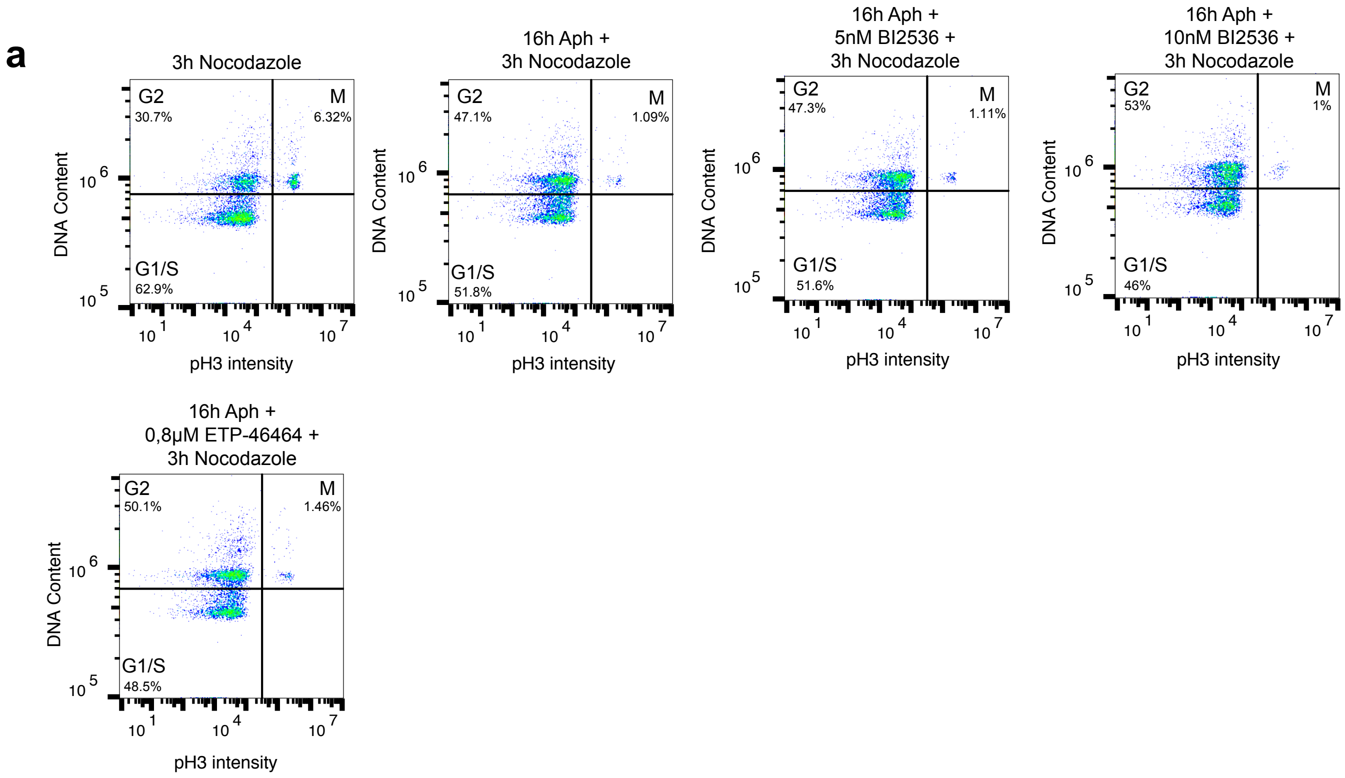

Supplementary Figure 4. Mild inhibition of Plk1 or ATR does not majorly change the cell cycle of RPE1 cells after replication stress: Cell cycle profile analysis by FACS of RPE1 cells stained with Propidium Iodide (DNA content marker) and phosho-H3 antibodies (mitosis marker) after 3 hours 1uM nocodazole, 16 hours 400 nM Aphidicolin + 3 hours nocodazole, 400 nM Aphidicolin + 5nM BI2536 + 3 hours nocodazole, 400 nM Aphidicolin + 10nM BI2536 + 3 hours nocodazole or 400 nM Aphidicolin + 0.8uM ETP-46464 + 3 hours nocodazole. The 3 hour nocodazole treatment served as a mitotic trap to reveal the number of cells entering in mitosis. The cell cycle phase of each population is marked on each panel.
