# Supplementary material for: Mild replication stress causes chromosome mis-segregation via premature centriole disengagement
Source: Nat Commun. 2019 Aug 8;10:3585. doi: 10.1038/s41467-019-11584-0 (PMC6687892; doi:10.1038/s41467-019-11584-0)
Supplement: Supplementary file 3 — Reporting Summary [file 41467_2019_11584_MOESM3_ESM.pdf]

nature research

Corresponding author(s): Patrick Meraldi
Therese Wilhelm

Last updated by author(s): Jul 3, 2019

Reporting Summary

Nature Research wishes to improve the reproducibility of the work that we publish. This form provides structure for consistency and transparency in reporting. For further information on Nature Research policies, see Authors & Referees and the Editorial Policy Checklist.

Statistics

For all statistical analyses, confirm that the following items are present in the figure legend, table legend, main text, or Methods section.

n/a	Confirmed	
☐	☒	The exact sample size (*n*) for each experimental group/condition, given as a discrete number and unit of measurement
☐	☒	A statement on whether measurements were taken from distinct samples or whether the same sample was measured repeatedly
☐	☒	The statistical test(s) used AND whether they are one- or two-sided<br>*Only common tests should be described solely by name; describe more complex techniques in the Methods section.*
☒	☐	A description of all covariates tested
☐	☒	A description of any assumptions or corrections, such as tests of normality and adjustment for multiple comparisons
☐	☒	A full description of the statistical parameters including central tendency (e.g. means) or other basic estimates (e.g. regression coefficient) AND variation (e.g. standard deviation) or associated estimates of uncertainty (e.g. confidence intervals)
☐	☒	For null hypothesis testing, the test statistic (e.g. *F*, *t*, *r*) with confidence intervals, effect sizes, degrees of freedom and *P* value noted<br>*Give P values as exact values whenever suitable.*
☒	☐	For Bayesian analysis, information on the choice of priors and Markov chain Monte Carlo settings
☒	☐	For hierarchical and complex designs, identification of the appropriate level for tests and full reporting of outcomes
☐	☒	Estimates of effect sizes (e.g. Cohen's *d*, Pearson's *r*), indicating how they were calculated

Our web collection on statistics for biologists contains articles on many of the points above.

Software and code

Policy information about availability of computer code

Data collection	The code for the microtubule depolymerization assay is available on the Switch platform.
Data analysis	Images were acquired and analyzed with NIS Elements software (Nikon), Imaris software (for 3D visualization), Matlab 2016a, FlowJo software (Tree Star), or SoftWorx (GE Healthcare). Statistics were evaluated with Prism 7.0 (GraphPad). Figures were mounted in Adobe Illustrator (Adobe)

For manuscripts utilizing custom algorithms or software that are central to the research but not yet described in published literature, software must be made available to editors/reviewers. We strongly encourage code deposition in a community repository (e.g. GitHub). See the Nature Research guidelines for submitting code & software for further information.

Data

Policy information about availability of data

All manuscripts must include a data availability statement. This statement should provide the following information, where applicable:

- Accession codes, unique identifiers, or web links for publicly available datasets
- A list of figures that have associated raw data
- A description of any restrictions on data availability

All data supporting the findings of this study are available within the article and its supplementary information files or from the corresponding author upon request. Due to their large size the primary and secondary data generated in the course of this project will be sent on external hard disks upon request.

Field-specific reporting

Please select the one below that is the best fit for your research. If you are not sure, read the appropriate sections before making your selection.

☒ Life sciences ☐ Behavioural & social sciences ☐ Ecological, evolutionary & environmental sciences

For a reference copy of the document with all sections, see nature.com/documents/nr-reporting-summary-flat.pdf

Life sciences study design

All studies must disclose on these points even when the disclosure is negative.

Sample size	All cells observed were included in the analysis. Otherwise sample size was chosen to fit the statistical test used.
Data exclusions	No data were excluded
Replication	Experiments were replicated at least three times.
Randomization	does not apply
Blinding	Blinding was used for cold stable experiments. All other experiments were analyzed by automated code or phenotypes were evident.

Reporting for specific materials, systems and methods

We require information from authors about some types of materials, experimental systems and methods used in many studies. Here, indicate whether each material, system or method listed is relevant to your study. If you are not sure if a list item applies to your research, read the appropriate section before selecting a response.

Materials & experimental systems

n/a	Involved in the study	
☐	☒	Antibodies
☐	☒	Eukaryotic cell lines
☒	☐	Palaeontology
☒	☐	Animals and other organisms
☒	☐	Human research participants
☒	☐	Clinical data

Methods

n/a	Involved in the study	
☒	☐	ChIP-seq
☐	☒	Flow cytometry
☒	☐	MRI-based neuroimaging

Antibodies

Antibodies used	rabbit anti α-tubulin (1/500, ab1851, abcam); mouse anti-CENP-F (1/1000, ab90, Abcam); mouse-GT335 (polyglutamylated tubulin, 1/2000, gift from Carsten Janke); γH2AX (1/2500, 05-636, Cell Signaling); 53BP1 (1/250, 4937, Cell Signaling); and CenpA antibodies (1/1000, ab13939, Abcam); Rat anti-Histone H3 tagged with AlexaFluor 647 (1/20, 558217, BD Bioscience); rabbit anti γ-tubulin (1/2000; this study)
Validation	Most antibodies are commercially available and/or have been previously published. More details can be found in the "methods" section under "immunofluorescence" or in the manufacturer's site. The rabbit anti γ-tubulin antibody was produced for this study as described in the "methods" section under "antibody production". The antibody was raised against a peptide that has been previously shown to result in high-affinity γ-tubulin antibodies (Fry et al., 1998). Moreover it was validated by immunofluorescence, as its staining pattern is the same as for other γ-tubulin antibodies.

Eukaryotic cell lines

Policy information about cell lines

Cell line source(s)	HCT116, Dld1, HT29 and H747 cells were obtained from Charles Swanton, hTert-RPE1 were obtained from ATCC, hTert-RPE1 Centrin1-GFP were obtained from Alexey Khodjakov and hTert-RPE1 EB3-GFP/H2B-RFP were obtained from Wilhelm Krek. Cal51, HCC1187 and HCC70 were obtained by ATCC and MCF10A were obtained from J.Curran lab.
Authentication	All cell lines used in this study were authenticated using STR profiling.
Mycoplasma contamination	We test all our cell lines for mycoplasma twice a year.
Commonly misidentified lines (See ICLAC register)	No

Flow Cytometry

Plots

Confirm that:

☒ The axis labels state the marker and fluorochrome used (e.g. CD4-FITC).

☒ The axis scales are clearly visible. Include numbers along axes only for bottom left plot of group (a 'group' is an analysis of identical markers).

☒ All plots are contour plots with outliers or pseudocolor plots.

☒ A numerical value for number of cells or percentage (with statistics) is provided.

Methodology

Sample preparation	Cells were pelleted, washed with PBS, fixed with 70% ice-cold ethanol, and stored at -20oC for at least 4 hours. After a wash with PBT- buffer (0.1g BSA and 5µl Tween in 10ml PBS), batches of 106 cells were incubated for 20min at RT with the mitotic marker Rat anti-Histone H3 tagged with AlexaFluor 647 (1/20, 558217, BD Bioscience).
Instrument	Labeled cells were detected with a Accuri C6 (BD) machine.
Software	Experiments were analyzed by FlowJo software (Tree Star).
Cell population abundance	At least 8000 events were recorded.
Gating strategy	First gating was done in order to record only single cells. This was done according to FS/SS scatters and PI-FL4 peak/ PI-FL4 area. Boundaries taken between positive and negative cells for each marker are shown in supplementary figure 1.

☒ Tick this box to confirm that a figure exemplifying the gating strategy is provided in the Supplementary Information.
